# Molecular Strategies for RPGR Gene Therapy

**DOI:** 10.3390/genes10090674

**Published:** 2019-09-04

**Authors:** Jasmina Cehajic Kapetanovic, Michelle E McClements, Cristina Martinez-Fernandez de la Camara, Robert E MacLaren

**Affiliations:** 1 Nuffield Laboratory of Ophthalmology, University of Oxford, Oxford OX3 9DU, UK; 2 Oxford Eye Hospital, Oxford University Hospitals NHS Foundation Trust, Oxford OX3 9DU, UK

**Keywords:** *Retinitis Pigmentosa GTPase Regulator*, gene therapy, adeno-associated viral, Retinitis Pigmentosa (RP)

## Abstract

Mutations affecting the *Retinitis Pigmentosa GTPase Regulator* (*RPGR*) gene are the commonest cause of X-linked and recessive retinitis pigmentosa (RP), accounting for 10%–20% of all cases of RP. The phenotype is one of the most severe amongst all causes of RP, characteristic for its early onset and rapid progression to blindness in young people. At present there is no cure for *RPGR*-related retinal disease. Recently, however, there have been important advances in *RPGR* research from bench to bedside that increased our understanding of *RPGR* function and led to the development of potential therapies, including the progress of adeno-associated viral (AAV)-mediated gene replacement therapy into clinical trials. This manuscript discusses the advances in molecular research, which have connected the RPGR protein with an important post-translational modification, known as glutamylation, that is essential for its optimal function as a key regulator of photoreceptor ciliary transport. In addition, we review key pre-clinical research that addressed challenges encountered during development of therapeutic vectors caused by high infidelity of the *RPGR* genomic sequence. Finally, we discuss the structure of three current phase I/II clinical trials based on three AAV vectors and *RPGR* sequences and link the rationale behind the use of the different vectors back to the bench research that led to their development.

## 1. Introduction

Inherited retinal diseases, most of which are retinitis pigmentosa (RP), affect 1 in 4000 people worldwide. The hallmark of this heterogeneous group of disorders is premature degeneration of rod and cone photoreceptors that leads to early vision loss. RP can be inherited as an autosomal recessive, dominant, X-linked, oligogenic, or mitochondrial trait. X-linked RP is one of the most severe forms of retinal degeneration and it accounts for 10%–20% of all RP cases [1,2,3]. To date, only 3 genes have been identified to be associated with X-linked pattern of inheritance. Mutations in the *Retinitis pigmentosa GTPase regulator* (*RPGR*) gene accounts for over 70% of X-linked RP cases whereas less common forms of the disease are caused by retinitis pigmentosa 2 (*RP2*) and 23 (*RP23* or *OFD1*) genes [4,5].

*RPGR*-related X-linked RP is characterised by severe disease in males with early onset and rapidly progressing sight loss that leads to legal blindness commonly by the fourth decade of life [2]. The classic rod-cone phenotype with peripheral pigmentary retinopathy, waxy optic disc pallor and vascular attenuation makes it often indistinguishable from other forms of RP. Less commonly, a cone-rod phenotype manifests with early central cone degeneration and accompanying loss of visual acuity. Female carriers of the *RPGR* disease are typically asymptomatic with a characteristic phenotype that manifests as a radial streak pattern originating from the fovea [6,7]. Rarely, however, skewed X-inactivation leads to more severe male-like phenotype with associated visual impairment [8].

At present, there is no approved treatment for retinitis pigmentosa caused by mutations in *RPGR*. Several treatment options have been under investigation and with the emergence of novel gene-based therapies for inherited retinal disease, this seems the most logical strategy to develop for the *RPGR* disease. Due to its severe phenotype, relatively high incidence and the fact that more commonly mutated genes such as *ABCA4* or *USH2A* are too large to be packaged into AAV vectors, the *RPGR* disease has drawn significant interest amongst scientific and clinical research communities over the last years. However, due to the inherent instability in the retina-specific RPGR^ORF15^ isoform sequence [9,10,11,12] the production of the therapeutic AAV-mediated *RPGR* vector has been very challenging. In attempts to improve the sequence stability and fidelity several approaches have been explored including codon optimisation [13,14,15], which has allowed generation of vectors for use in human trials. In this review we discuss recent advances in the understanding of *RPGR* gene structure and its evolutionary conservation that has led to an improved understanding of protein’s molecular function and mechanisms implicated in the pathogenesis of RPRG-related retinal dystrophy. The pre-clinical development of gene therapy vectors that has resulted in their progression into three phase I/II clinical trials is covered in detail, including discussion on three different *RPGR* cDNA sequences used in the trials.

## 2. Structure and Function of *Retinitis Pigmentosa GTPase Regulator (RPGR)*

**Figure 1:**
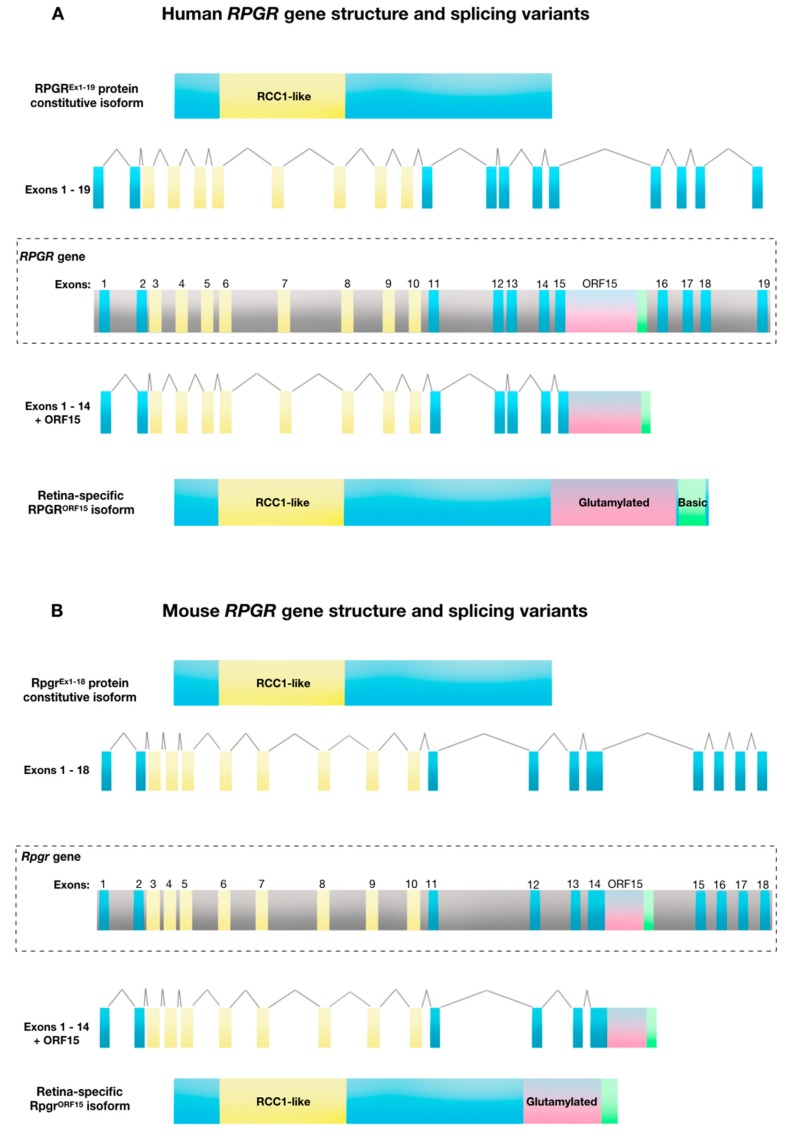
*Retinitis Pigmentosa GTPase Regulator (RPGR)* gene structure and splicing variants. (**A**) Human *RPGR* gene exon-intron structure showing the combination of exons 1 to 19 to create the constitutive protein isoform, and alternative splicing of exon 15/intron 15 that creates the RPGR^ORF15^ variant. (**B**) Mouse RPGR gene exon-intron structure showing the combination of exons 1 to 18 to create the constitutive protein isoform and alternative splicing of intron 14 creates the RPGR^OFR15^ variant.

The human *RPGR* gene is located on the short arm of the X-chromosome (Xp21.1). The gene exhibits a complex expression pattern with 10 alternatively spliced isoforms, five of which are protein coding [16]. The first transcript to be identified in association with X-linked retinitis pigmentosa, was the constitutive RPGR^Ex1-19^ isoform. In humans, the RPGR^Ex1-19^ isoform contains 19 exons and expresses a full-length messenger RNA transcript of 2448 bp, which generates an 815 amino acid sequence that forms ~90 kDa protein in a variety of tissues [17]. Since this initial characterisation, multiple alternative transcripts have been identified, including the retina-specific RPGR^ORF15^ variant [10,16,18]. This variant contains exons 1–14 of constitutive RPGR with the exon ORF15 derived from alternatively spliced exon 15 and intron 15 (Figure 1A). The RPGR^ORF15^ isoform is 3459 bp, encoding a 1152 amino acid sequence which forms a ~200 kDa protein. As with the widely expressed variant, amino acids 54–367 (exons 3–10) form a regulator of chromosome condensation 1 (RCC1)-like domain. The alternative ORF15 exon consists of a highly repetitive purine-rich sequence coding for multiple acidic glutamate-glycine repeats. This is followed by a C-terminal tail region rich in basic amino acid residues, called the basic domain.

The reason for this complex expression pattern of the RPGR protein remains largely unknown, but may be related to the functional role of its splice isoforms in various cell types. The RPGR protein is widely expressed in vertebrate tissue including eye, brain, lung, testis and kidney. In the eye, the two major isoforms, RPGR^Ex1-19^ and RPGR^ORF15^ are predominantly localised to the photoreceptor connecting cilia [19] and less consistently, to the nuclei and photoreceptor outer segments of some species [20]. The connecting cilium is a critical junction between the inner and outer photoreceptor segments, controlling the bidirectional transport of opsin and other proteins involved in the phototransduction cascade and the overall health and viability of the photoreceptors. Attempts are ongoing to elucidate further the expression patterns of RPGR through evolutionary characterisation of RPGR domains across species and via molecular interactions of RPGR with other proteins in order to shed light on the exact role of the RPGR protein.

The RCC1-like domain, present in both major splice forms, adopts a seven-bladed β-propeller structure and it is strongly conserved across evolution, in vertebrates and invertebrates [9]. This domain has been implicated in a regulatory role of small GTPases. It is thought to enable RPGR to act as a Ran guanine nucleotide exchange factor and RPGR has been shown to upregulate the guanine nucleotide exchange factor RAB8A, associating with the GDP-bound form of RAB8A to stimulate GDP/GTP nucleotide exchange [21]. The RCC1-like region also interacts with: RPGR interaction protein 1 (RPGRIP1), which links it to the connecting cilium of photoreceptor cells [19]; the lipid trafficking protein phosphodiesterase 6D (PDE6D) [22]; two chromosome-associated proteins important for the structural maintenance of chromosomes, SMC1 and SMC3 [23] and two ciliary disease-associated proteins nephrocystin-5 (NPHP5) [24] and centrosomal protein 290 (CEP290) [25].

The retina-specific ORF15 domain is also highly evolutionarily conserved across varied species, indicating a functional importance (Table 1). However, in contrast to the RCC1-like domain, the ORF15 domain is unique to vertebrates, suggesting a role that is unique to the ciliary-derived photoreceptors of “simple” vertebrate eyes, compared to the rhabdomeric photoreceptors of “compound” invertebrate eyes. Hence, the ciliary-based transport of cargoes such as rhodopsin, which is at least 10 times more abundant in vertebrates than invertebrates, fits with this hypothesis. ORF15 homology and a region of high AG content of >80% is identifiable in a range of species although the length varies—the mouse ORF15 is shorter than the human ORF15, Figure 1A). This purine-rich region of ORF15 (97.5% purines within 1kb in humans) encodes the glutamine-glycine rich domain that ends in a basic C-terminal domain, which is also highly conserved, suggesting that it constitutes another functional region. This basic domain, which is unique to RPGR^ORF15^, interacts with at least two proteins, a chaperone protein nucleophosmin and a scaffold protein whirlin [26]. Neither protein is unique to vertebrate photoreceptors, but nucleophosmin is present in metaphase centrosomes during cell division, while whirlin helps to maintain ciliary structures within the eye and ear. 

The function of the repetitive glutamine-glycine-rich domain itself has been difficult to establish due to its variable length and relatively poor conservation at the individual amino acid level, although the overall charge and repeat structure length remain conserved in vertebrates. However, recent evidence shows that this intrinsically disordered region is heavily glutamylated [27], a post-translational protein modification that adds glutamates to target proteins to affect their stabilisation and folding. This process is known to be essential for the function of tubulins in intracellular trafficking [28]. Furthermore, this glutamylation has been shown to be achieved by tubulin tyrosine ligase like-5 (TTLL5) enzyme, which interacts directly with the basic domain of the OFR15 to bring it into the proximity of glutamylation sites along the glutamine-glycine-rich repetitive region [29]. The role of the ORF15 region is of course critically important to photoreceptor function, because otherwise ORF15 mutations would not be pathogenic since the RPGR^EX1-19^ variant is still expressed in these cells. Hence in-frame deletions in the ORF15 region lead to progressive loss of function as the deletion length increases [13].

## 3. Molecular Mechanisms and Pathogenesis of RPGR-Related X-Linked Retinitis Pigmentosa (RP)

Molecular mechanisms and pathogenesis of RPGR-related X-linked RP have been under investigation for several decades. The drive to better understand the disease process comes from the high incidence with mutations in the gene encoding RPGR accounting at least 70% of X-linked RP and up to 20% of all RP cases [2,3,4]. Moreover, the disease is associated with one of the most severe phenotypes among inherited retinal diseases with central visual loss occurring early in adult life [2]. This coupled with the developments in genetic therapies has given impetus to a large number of studies aimed to uncover the pathogenic mechanisms.

Despite ubiquitous expression of the constitutive RPGR variant in ciliated cells throughout the body, the RPGR^Ex1-19^ has yet to show a firm association with any human disease. The RPGR-related phenotype seems to be confined to the retina and several studies have established an essential role for RPGR^ORF15^ in photoreceptor function and survival [10,11]. Genetic studies have shown that mutations in the RPGR^ORF15^ result in abnormal protein transport across the connecting cilium, which can lead to photoreceptor cell death [12,30,31]. However, there are reports in the literature that describe RPGR-related X-linked retinitis pigmentosa syndrome comprising of retinitis pigmentosa, recurrent respiratory tract infections and hearing loss [32,33]. These findings point to the abnormalities in respiratory and auditory cilia in addition to the photoreceptors. In addition, as photoreceptors develop from ciliated progenitors, it has been postulated that the axoneme may play a role in their early development. Sperm axonemes were thus studies in patients with X-linked retinitis pigmentosa and a significant increase in abnormal sperm tails was observed [34]. Similar findings have been reported in another syndromic ciliopathy, the Usher syndrome [35].

Mutations in *RPGR* account for ~70% of cases of X-linked RP and have been identified across exons 1–15, yet up to 60% of mutations occur in the ORF15 region [10,30]. The repetitive nature of the glutamate-glycine region in ORF15 is prone to adopt unusual double helix DNA conformations or triplexes that are thought to promote polymerase arrest and block replication and transcription. These imperfections are likely to contribute to genome instability and account for the high frequency of mutations in this region, known as the mutation ‘hot spot’ of the *RPGR.* Surprisingly, no disease-causing mutations have been reported in exons 16–19 [36].

The most common mutations are small deletions that lead to frameshifts followed by nonsense mutations [30]. Within ORF15, the most common mutations are microdeletions 1–2, or 4–5 bp [10], that cause frameshifts leading to truncated forms of the protein and in particular, loss of the C-terminus. Small in-frame deletions or insertions (and missense changes) that can alter the length of ORF15 region by a few base-pairs (e.g., up to 36, equivalent to 9 amino acids in this population based study [37], are seemingly well tolerated [38]. Thus, despite being a coding region, this domain has a surprisingly high rate of tolerable indels within primate lineages, suggesting a rapidly evolving region [9]. However, recent evidence shows that larger deletions in the ORF15 region significantly affect the degree of RPGR glutamylation, which may subsequently influence its function and ability to associate with the cilium and other interacting factors [29]. Thus, frame shift mutations that lead to loss of the C-terminal basic domain are invariably disease causing [12]. In addition, mutations that lead to the loss of TTLL5 enzyme, the basic domain-binding partner that mediates RPGR glutamylation, abort glutamylation process and cause RPGR-like phenotype in humans [39]. This further supports the critical role of glutamylation in normal RPGR^ORF15^ function. It remains intriguing that despite its ubiquitous expression, the RPGR^Ex1-19^ is unable to compensate for the loss of function of RPGR^ORF15^ in the retina to rescue the phenotype. It is possible that the alternative splicing in the retina could favour the RPGR^ORF15^ variant, so the majority of transcripts will be the RPGR^ORF15^ isoform, with few constitutive variants available to compensate. One study failed to identify the constitutive transcript in the retina [18], which supports the finding that the constitutive isoform is expressed early in development in a mouse before its levels decline and the RPGR^ORF15^ becomes the predominant isoform [26]. Notably, the constitutive variant lacks the glutamate-glycine repetitive region and given the importance of this domain for the normal function of RPGR^ORF15^ in the photoreceptors, perhaps it is not so surprising that the constitutive variant cannot offer the same functional benefit as the RPGR^ORF15^ variant.

## 4. Clinical and Genetic Diagnosis of RPGR-Related X-Linked RP

The diagnosis of RPGR-related retinal dystrophy is made on the basis of presenting symptoms and retinal signs seen on clinical examination and various imaging modalities. In addition, study of family history showing X-linked inheritance (no male to male transmission) and genetic testing identifying the pathogenic mutation are important in confirming the diagnosis. In cases of uncertain diagnosis and unequivocal genetic test results we have adopted several important steps, which are discussed below, in order to minimise the risk of establishing an incorrect diagnosis, and administering the patient with an incorrect gene if recruited into a gene therapy clinical trial.

**Figure 2:**
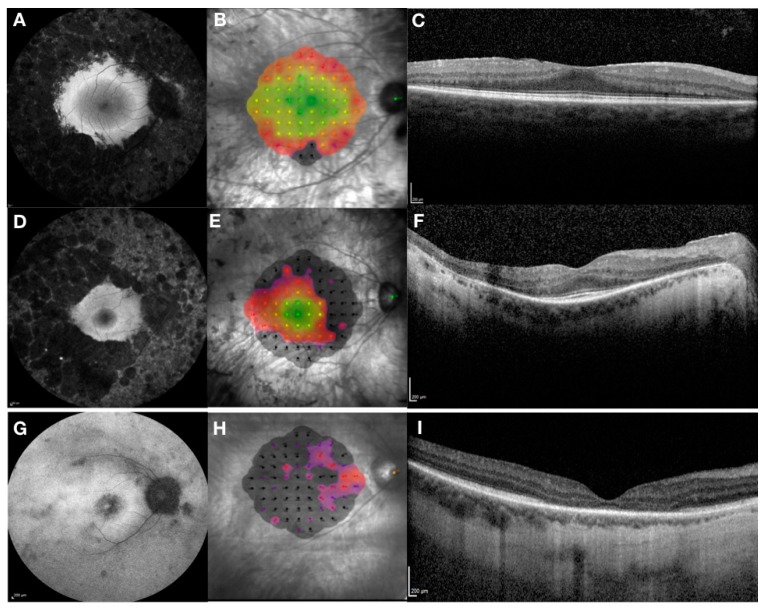
Clinical phenotypes associated with *RPGR* retinal degeneration—rod-cone phenotype (early stage (**A**–**C**) and a more advanced stage (**D**–**F**)) and cone-rod phenotype (**G**–**I**). The phenotypes are captured by Heidelberg fundus autofluorescence, (left column), MAIA microperimetry measuring central retinal sensitivity (central column; sensitivity is represented by a heat map: green/yellow—normal/mildly reduced; red/purple—reduced; black—not measurable) and Heidelberg optical coherence tomography showing retinal structures in cross-section (right column). In rod-cone phenotype there is extensive peripheral retinal atrophy with relative preservation of central retina as seen on autofluorescence associated with para-foveal hyper-autofluorescent ring (**A**). This is confirmed by near normal central retinal sensitivity (**B**) and preservation of ellipsoid zone (**C**). In more advanced stages of the disease there is reduction in size of the para-foveal hyper-autofluorescent ring (**D**) with corresponding reduction in retinal sensitivity (**E**) and length of ellipsoid zone (**F**). In contrast, in cone-rod phenotype there is early loss of para-foveal photoreceptors with associated hypo-fluorescent ring and marked reduction of retinal sensitivity with corresponding loss of the ellipsoid zone.

RPGR-related retinal dystrophy is associated with a very heterogeneous phenotype that ranges from pan-retinal rod-cone to predominant cone dystrophy (Figure 2). The phenotype is generally more severe with faster progression compared to other forms of RP and median age of legal blindness of approximately 45 years old, which is much younger than in other RP genotypes [40]. Most patients lose their peripheral vision first, followed by the loss of central vision. Recent evidence suggests that the rod-cone phenotype is found in 70% of patients, the cone-rod in 23% and the cone phenotype in 7% of patients with X-linked RPGR related retinal dystrophy [2]. The study shows that the onset of symptoms was in early childhood in rod-cone dystrophy (median age 5 years) and in third decade in cone-rod and cone dystrophy, although the age range was very wide (between 0 and 60 years). However, cone-rod and cone dystrophies were associated with a more severe phenotype and the probability of being blind at the age of 40, with visual acuity of less than 0.05 LogMAR (3/60 or 20/400) observed in 55% of patients with cone-rod and cone dystrophy compared to only 20% in rod-cone dystrophy.

The RPGR phenotype (Figure 2) has been associated with anatomical changes including central retinal thinning of the outer nuclear layer as seen on retinal cross-sections taken by optical coherence tomography [40,41]. The junction between the inner and outer photoreceptor segments, better known as the ellipsoid zone, can be used as an important predictor of central retinal function and for monitoring of disease progression. [42]. Thus, the disruption of the ellipsoid zone can be detected with corresponding early reduction in visual acuity and retinal sensitivity as measured by microperimetry. In addition, autofluorescence can be used to assess the health of the retinal pigment epithelium with early signs of hyper-autofluorescence indicating accumulation of lipofuscin and related metabolites as a by-product of photoreceptor outer segment degradation. Later in the disease process, areas of hypo-autofluorescence become evident indicating outer retinal atrophy with loss of retinal pigment epithelium cells. The RPGR phenotype is often associated with para-foveal hyper-autofluorescent rings, which decline exponentially with disease progression [43]. Constriction areas are correlated highly with baseline area and age, where younger subjects had greatest rate of progression. No correlation with genotype was observed in this study. In the cone-rod phenotype, however, the area of hypo-autofluorescence associated with a surrounding hyper-autofluorescent ring tends to increase in size with disease progression and is inversely related to electroretinogram amplitude [44]. Ongoing natural history studies are promising to shed more light on the natural progression of the RPGR disease phenotypes and provide better understanding and interpretation of clinical trial endpoints used in current interventional gene therapy trials (Table 2).

**Figure 3:**
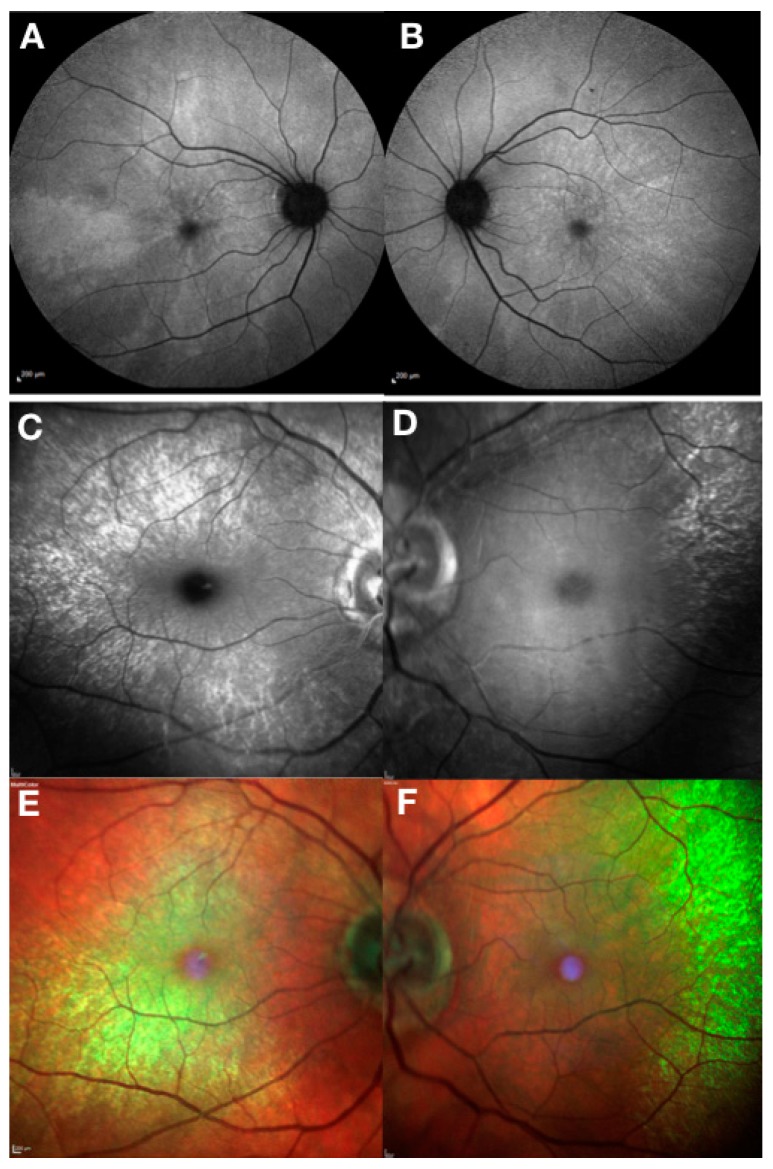
**Clinical phenotype of *RPGR* female carriers.** Fundus autofluorescence (Heidelberg) showing a typical macular radial pattern or ‘tapetal’ reflex in a female carrier of an *RPGR* mutation (**A**,**B**). Random X-chromosome inactivation generates clones of normal or affected photoreceptors giving rise to this mosaic pattern. Blue reflectance (**C**,**D**) and multicoloured (**E**,**F**) modes using Heidelberg scanning laser ophthalmoscope can be very helpful in showing the macular reflex.

Female carriers of *RPGR* mutations also show high phenotypic variability [7] (Figure 3). The carrier phenotype includes asymptomatic females with near-normal clinical appearance, macular pattern reflex with different degrees of pigmentary retinopathy and severely affected females with clinical phenotype that results from skewed X chromosome inactivation and is indistinguishable from the male pattern. Female carriers with male pattern dystrophy should be considered for *RPGR* gene therapy as discussed below.

The molecular diagnosis using next-generation sequencing (NGS) is usually a robust approach in determining pathogenic variants in RP. However, the ORF15 region of RPGR is not normally sequenced with NGS methods and is currently only performed upon specific request. Moreover, sequencing of the ORF15 region in *RPGR* is notoriously difficult and error-prone. Overlapping reading frames and polymorphic deletions/insertions add further complexity to the detection of true mutations. Additional precautions must, therefore, be taken with interpreting the sequencing data so that small deletions are not confused with artefacts that would lead to spurious results. In cases of uncertainty, testing should be repeated. In addition, the full RP panel should be performed to exclude other pathogenic variants including the sequencing of *RP2* and *OFD1* X-linked genes. This comprehensive molecular genetic analysis together with the *RPGR* phenotype and a clear family history of X-linked inheritance, including evidence of a carrier phenotype, forms the basis of inclusion criteria into gene therapy clinical trials. In addition, a recent study describes an in vitro assay for determining the pathogenicity of *RPGR* missense variations [45]. The strategy is based on the RPGR protein interaction network, which is disrupted by missense variations in RCC1-like domain in RPGR, and could help to differentiate between causative missense mutations and non-disease-causing polymorphisms.

## 5. Treatment Options for RPGR-Related X-Linked RP

Several non-gene based treatment approaches have been investigated for the preservation of vision in X-linked RP including a nutritional supplement, docosahexaenoic acid [46] and a ciliary neurotrophic factor [47] both of which were unable to prevent photoreceptor degeneration and visual loss. For patients with advanced disease, electronic retinal devices have demonstrated proof-of-concept in their ability to restore crude vision [48,49]. However, the unpredictability of benefit for individual patients and the high price of these devices make it economically difficult to maintain their availability for the treatment of patients with RP. Another potential strategy, optogenetics, is under investigation and has shown promising results for vision restoration in advanced retinal degeneration [50,51].

Emerging gene-based therapy using the AAV vector is currently the most promising therapeutic strategy for RPGR X-linked RP. The size of the coding sequence of RPGR^ORF15^ (3.5 kb) is within the AAV carrying capacity and the relatively high prevalence and disease severity have justified development of this therapy. However, the repetitive sequence of ORF15 not only makes it a hotspot for mutations but also creates challenges for therapeutic vector production. Attempts to generate AAV vectors for RPGR gene-supplementation strategies have been thwarted by the poor sequence stability of the ORF15 region and transgene production has struggled to control spontaneous mutations and maintain the complete sequence [13,52,53,54,55]. AAV gene therapy in two RPGR X-linked RP canine models that carry different ORF15 mutations [55] provided proof of concept for treating RPGR mutations within the ORF15 region. AAV2/5-mediated sub retinal gene delivery of a full-length human RPGR-ORF15 cDNA [10], driven by either the human interphotoreceptor retinoid-binding protein (hIRBP) promoter or the human G-protein-coupled receptor kinase 1 (hGRK1) promoter, prevented photoreceptor degeneration and preserved retinal function in both canine models. However, the AAV2/5.RPGR vector was found to have multiple mutations within the purine-rich exon 15 region that led to toxic effects in mice at higher doses [52] thus posing safety questions for human applications. In an attempt to improve the sequence stability, a step-wise cloning approach was used to generate the correct full-length RPGR^ORF15^ coding sequence (the purine-rich region was generated first and then ligated to the rest of the DNA sequence) [53], which was packaged into the AAV8.GRK1.*RPGR^ORF15^* vector and evaluated in the *Rpgr*-KO mouse. However, despite improved stability, some vector preparations were still ridden with micro-deletions that led to expression of alternatively spliced truncated forms of the RPGR protein that was mislocalised to photoreceptor inner segments and only a partial rescue of the phenotype in treated mice. The truncated forms of the protein were further investigated for their ability to rescue the RPGR phenotype in the *Rpgr*-KO mouse [13]. The short (314 out of 348 ORF15 codons deleted) and the long (126 out of 348 codons deleted) forms of the *RPGR^ORF15^* were tested. The long form demonstrated significant improvement in the disease phenotype, whilst the short form failed to localise correctly in the photoreceptors and showed no functional rescue of the phenotype. Importantly, as discussed above, large deletions in the ORF15 region can affect the glutamylation of the protein and lead to impaired function. Indeed, a follow-up study by the same group tested these truncated vectors [29] for their glutamylation capacity. Unsurprisingly, the long form demonstrated significantly impaired glutamylation (only 30% of the full length protein), whereas the short form showed no detectable glutamylation of the RPGR protein.

To circumvent these issues, the research team of Fischer and colleagues (2017) generated a full-length, human, codon-optimised version of RGPR^ORF15^ to stabilise the sequence, remove cryptic splice sites and increase expression levels from the therapeutic transgene [14]. This enabled reliable cloning and vector production. The resulting AAV8.coRPGR^ORF15^ vector was shown to offer therapeutic rescue in two mouse models of X-linked RP (*Rpgr^-/y^* and *Rd9*). This vector is now being used in a Phase I/II/III gene therapy clinical trial in humans (NCT03116113). In addition, the codon optimised form of the RPGR vector used in the canine studies [15] and the truncated form of the RPGR with near-total OFR15 deletion [13] are also being tested in ongoing clinical trials (NCT03316560 and NCT03252847 respectively) as will be discussed further in the next section. A very recent study used a bioinformatics approach as an alternative method to develop a molecularly stable *RPGR* gene therapy vector [56]. The strategy identified regions of genomic instability within ORF15 and made synonymous substitutions to reduce the repetitive sequence and thus increase the molecular stability of *RPGR*. The codon optimized construct was validated in vitro in pull-down experiments and in a murine model, demonstrating production of functional RPGR protein.

## 6. Gene Therapy Clinical Trials for RPGR-Related X-Linked RP

**Figure 4:**
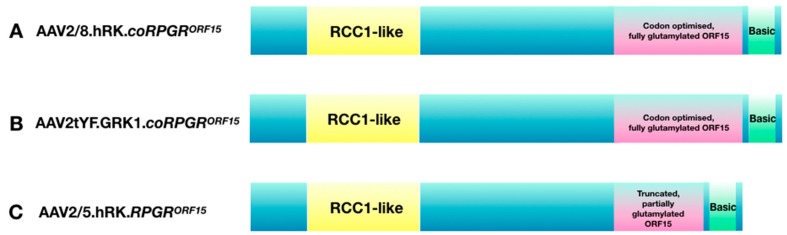
**AAV vector constructs used in current gene therapy trials:** (**A**) the Nightstar Therapeutics (now Biogen Inc) trial, NCT03116113; (**B**) the Applied Genetic Technologies Corporation trial, NCT03316560; (**C**) the MeiraGTx trial, NCT03252847.

The results of the pre-clinical studies described above support the use of AAV-based gene therapy for RPGR-related X-linked RP in humans, in the early to mid-stage of the disease. Ideally, patients with moderately reduced visual acuity and constricted visual fields, but a preserved central ellipsoid zone, should be recruited into gene therapy trials for best expected therapeutic benefits. Interestingly, development of RPGR therapy from bench to bedside has resulted in setting-up of three multi-centre dose-escalation gene-therapy clinical trials (see Table 2 for details). Each trial is using a different combination of AAV vector variant and *RPGR* coding sequence (Figure 4). Specifically, the Nightstar Therapeutics (now Biogen Inc) sponsored trial (NCT03116113) is using the wild-type AAV8 vector with a human rhodopsin kinase promoter and a human codon optimised full-length RPGR^ORF15^ cDNA sequence (AAV2/8.hRK.*coRPGR^ORF15^*). The second trial sponsored by Meira GTx (NCT03252847) is using a wild-type AAV2/5 capsid with a truncated, non-codon optimised *RPGR* sequence under control of the human rhodopsin kinase promoter (AAV2/5.hRK.*RPGR^ORF15^*). The third trial conducted by Applied Genetic Technologies Corporation (NCT03316560) is using mutated AAV2 capsids (capsids with single tyrosine to phenylalanine (YF) mutations) packaged with full-length, codon optimised human *RPGR^ORF15^* sequence also driven by the rhodopsin kinase promoter (AAV2tYF.GRK1.*coRPGR^ORF15^*).

The pre-clinical studies that led to the development of vectors used in human trials were described in detail in the previous section. However, the rationale for using the three different vectors deserves further discussion. The coding sequence used in the Meira GTx trial is an abbreviated form of human *RPGR^ORF15^* sequence. The rationale provided for using the truncated form, which arose through a spontaneous mutation resulting in deletion of one third of the ORF15 region, was because the deletion led it to become more stable, thereby reducing the rate of further recombination errors and potential mutations. Interestingly, the authors also showed that further shortening of this critical ORF15 region significantly affects the protein function, leading to mislocalisation of the protein in photoreceptors and no functional or morphological rescue in a mouse model, confirming the importance of the ORF15 region for photoreceptor function. Importantly, a further study demonstrated that the post-translational glutamylation is reduced by over 70% in this abbreviated form of the RPGR^ORF15^, significantly affecting trafficking of molecules critical for photoreceptor function [29]. However, since RPGR is not expressed highly in photoreceptors, it is possible that over-expression of RPGR with gene therapy can compensate for the reduced trafficking ability. The truncated construct was shown to rescue the photoreceptor function in a murine model of X-linked RP [13]. However, the mouse *RPGR^ORF15^* is naturally shorter than the human *RPGR^ORF15^* with an abbreviated ORF15 region (see Figure 2 and Table 1) much like the engineered abbreviated human construct used in the human trial. Thus, it may not be so surprising that the abbreviated human construct led to the rescue in a murine model, as the two sequences are very similar and the murine model has a milder phenotype compared to humans. The efficacy of this shortened version of *RPGR^ORF15^* has not been evaluated in canine models of X-linked RP and the results from human trials are awaited in anticipation.

The constructs used in the AGTC and the Nightstar Therapeutics (now Biogen Inc.) trials are very similar and encode the full-length human wild-type RPGR^ORF15^ protein. Both constructs applied codon optimisation that was shown by Fischer et al. to confer greater sequence stability with higher expression levels than wild-type RPGR sequence, whilst not affecting the glutamylation pattern in the RPGR protein. The codon-optimised RPGR rescued the disease phenotype in two mouse models of X-linked RP [14] and was recently also validated in the RPGR canine model [15] showing transduction of both rods and cones and preserving the outer nuclear layer structure in the treated retina. The results of the phase I/II trials are expected in the near future.

## 7. Summary

X-linked RPGR-related RP is a heterogenous group of disorders with no clear genotype–phenotype correlation. Both rod-cone and cone-rod retinal dystrophies are seen with relatively early onset and rapid progression to blindness that is related to mutations that cause loss of function of this key photoreceptor protein. The complex expression pattern of the *RPGR* gene through cryptic splice sites that create multiple isoforms poses challenges in elucidating its function. However, mounting evidence suggests that retina-specific RPGR^ORF15^ is unique to vertebrates and plays a crucial role in regulating protein trafficking between inner and outer segments as well as in microtubular organisation. Importantly, RPGR^ORF15^ contains a characteristic repetitive purine-rich region that is highly glutamylated and only the glutamylated RPGR^ORF15^ is fully functional. Thus, any mutations that reduce the glutamylation process adversely affect RPGR protein function. In addition, the ORF15 region created challenges for the researches interested in developing *RPGR* gene-based therapies as the repetitive region made it unstable and prone to mutations. The current approach in developing a codon-optimised version of the RGPR^ORF15^ to stabilise the sequence, remove cryptic splice sites and increase expression levels from the therapeutic transgene is now being used in humans, following proof-of-concept studies in murine and canine models of X-linked RP. This approach has allowed the rapid progression towards the first in-human gene therapy trial (NCT03116113) for X-linked RP, which began in March 2017. In parallel, two additional independent research consortia have been developing gene therapies for the RPGR disease. With recent approval of gene replacement therapy Luxturna, for the treatment of *RPE65*-related retinal disease, the precedence for approval of future gene-based therapies has been set and results of the RPGR early phase clinical trials are awaited with great expectation.

## Figures and Tables

**Table 1:** **Evolutionary conservation of DNA and amino acid sequences of RPGR^ORF15^ variants across selected species.** All data were extracted from NCBI database files with comparisons performed in Geneious Prime 2017.10.2. For *Homo sapiens*, details were extracted from gene files NG_009553.1 and 6103 combined with mRNA file NM_001034853.2. * The conserved basic domain of the human *RPGR^ORF15^* coding sequence was used for predictions of ORF15 locations in all other species sequences by homology alignment. For *Mus musculus* data, gene files NC_000086.7 and 19893 were aligned with the basic domain of human *RPGR^ORF15^* and the partial sequence file AF286473.1 to identify the predicted ORF15 variant. For *Canis lupus familiaris* data, gene files 403726 and AF148801.1 were aligned with the basic domain of human *RPGR^ORF15^* and the partial sequence file AF385629.1. For *Pan troglodytes* data, files 4465569 and XM_024352988 were used. For *Gorilla gorilla gorilla*, files 101149059, the basic domain of human *RPGR^ORF15^* and the partial sequence AY855163.1 were combined. For *Macaca mulatta*, files 714316, the basic domain of human *RPGR^ORF15^* and the partial sequence file AY855162.1 were combined. Finally, *Xenopus tropicalis* sequence predictions were achieved from files 733454 and XM_018091818.1.

	DNA Sequence				Amino Acid Sequence	
Species	Coding Sequence Prior to ORF15	Percentage of Purine Bases	Region with Homology to Human ORF15 *	Percentage of Purine Bases	ORF15 Amino Acid Length (Percentage Glu-Gly) *	Glutamylation Region (Percentage Glu-Gly) *
***Homo sapiens***	1 to 14	54%	ORF15	89%	567 (67%)	351 (88%)
	1.7 kb		1.7 kb			
***Mus musculus***	1 to 14	57%	Intron 14	86%	488 (60%)	273 (84%)
	2.5 kb		1.5 kb			
***Canis lupus familiaris***	1 to 13	58%	Exon 14/Intron 14	88%	522 (66%)	331 (72%)
	2.5 kb		1.5 kb			
***Pan troglodytes***	1 to 14	54%	Exon 15/Intron 15	89%	560 (66%)	330 (88%)
	1.7 kb		1.7 kb			
***Gorilla gorilla gorilla***	1 to 14	54%	Exon 15/Intron 15	89%	549 (66%)	321 (88%)
	1.7 kb		1.7 kb			
***Macaca mulatta***	1 to 14	53%	Exon 15/Intron 15	89%	549 (65%)	323 (86%)
	1.7 kb		1.7 kb			
***Xenopus tropicalis***	1 to 13	57%	Exon 14/Intron 14/Exon 15	77%	679 (45%)	232 (82%)
	1.6 kb		2.0 kb			

**Table 2:** Summary of clinical trials for RPGR-related X-linked retinitis pigmentosa (RP).

Clinical Trial (clinicaltrials.gov)	Intervention/Observation	Clinical Centre/s	Sponsor
Phase I/II/III NCT03116113 multicenter, open-label Part 1: non-randomised, dose-selection study 18 participants Part 2: dose expansion study (randomised to low dose, high dose, control) 63 participants Start date: March 2017	Subretinal delivery of AAV8-hRK-coRPGR^ORF15^	Oxford, UK Manchester, UK Southampton, UK Florida, USA Oregon, USA Pennsylvania, USA	Nightstar Therapeutics (now Biogen Inc), UK
Phase I/II NCT03252847 Non-randomised, open-label, dose-escalation trial 36 participants Start date: July 2017	Subretinal delivery of AAV2/5-hRK-RPGR^ORF15^	London, UK	MeiraGTx, UK
Phase I/II NCT03316560 Non-randomised, open-label, multicenter, dose-escalation trial 30 participants with RPGR ORF15 mutations Start date: April 2018	Subretinal delivery of rAAV2tYF-GRK1-coRPGR^ORF15^	Colorado, USA Massachusetts, USA New York, USA North Carolina, USA Ohio, USA Oregon, USA Pennsylvania, USA Texas, USA	Applied Genetic Technologies Corporation (AGTC), USA
Prospective natural history study of XLRP with genetically confirmed mutation in RPGR 150 participants Start date: December 2017	Observational study	Multiple centres in UK, Germany, Holland, France, USA	Nightstar Therapeutics (now Biogen Inc), UK
Prospective natural history study of XLRP NCT03349242 Start date: December 2017	Observational study	Massachusetts, USA Michigan, USA	MeiraGTx, UK
Prospective natural history study of XLRP caused by RPGR-ORF15 mutations 45 participants NCT03314207 Start date: December 2017	Observational study	New York, USA North Carolina, USA Ohio, USA Oregon, USA Texas, USA	Applied Genetic Technologies Corporation (AGTC), USA
